# Narrative Review of the Role of Patient-Reported Outcomes and Inhaler Handling Errors in the Control of Asthma and COPD

**DOI:** 10.1007/s11882-022-01041-2

**Published:** 2022-09-10

**Authors:** Raúl De Simón Gutiérrez, Raúl Piedra Castro

**Affiliations:** 1 Primary Care Physician, Luis Vives Health Center, Alcala de Henares, Madrid, Spain; 2 Primary Care Physician, Henares Azuqueca Health Center, Guadalajara, Spain

**Keywords:** Asthma, COPD, Inhaler error, Patient-reported outcomes, Dry powder inhalers, Spiromax

## Abstract

**Purpose of Review:**

Asthma and chronic obstructive pulmonary disease (COPD) are chronic respiratory diseases that remain uncontrolled in many patients, despite the wide range of therapeutic options available. This review analyzes the available clinical evidence on 3 budesonide/formoterol DPI devices, Spiromax^®^, Turbuhaler^®^, and Easyhaler^®^, in terms of patient-reported outcomes (PROs), inhaler errors, and asthma and COPD control.

**Recent Findings:**

The effectiveness of dry powder inhalers (DPI) depends largely on the device and the patient’s inhaler technique. Equally important are the patient’s perception of the inhaler and adherence. Given the high burden of these diseases, it is important that efforts be made to select the best DPI for each patient and to analyze the impact of these variables to help improve the health and quality of life of our patients.

**Summary:**

This review provides a comprehensive overview of the present knowledge about PROs, inhaler handling errors, and asthma and COPD control achieved by Spiromax^®^, Turbuhaler^®^, and Easyhaler^®^.

## Introduction

Asthma and chronic obstructive pulmonary disease (COPD) are major causes of morbidity and mortality worldwide [1]. Both diseases confer a significant burden on patients and their families and on healthcare systems [2, 3].

Treatment is based on the delivery of drugs via an inhaler, with the aim of controlling symptoms, reducing exacerbations, improving exercise tolerance and health status, and reducing mortality [4]. The therapeutic options recommended in clinical practice guidelines for patients with asthma and COPD include various fixed-dose combinations of an inhaled corticosteroid (ICS) and a long-acting beta-adrenergic agonist (LABA). One commonly used ICS/LABA combination is budesonide and formoterol (BF). BF is delivered via Spiromax^®^, Turbuhaler^®^, and Easyhaler^®^ dry powder inhalers (DPIs) in the management of asthma and COPD.

Although many pharmacological treatments are recommended in clinical practice guidelines, both diseases remain uncontrolled in a considerable number of patients [5, 6]. This is because the effectiveness of the drugs administered via DPIs depends on the patient’s inhaler technique [7], and this effectiveness is significantly diminished when patients fail to manage their inhaler correctly [2, 3, 8, 9]. Similarly, the patient’s perception of their inhaler and their therapeutic adherence, which are of particular importance in the setting of a chronic disease, can greatly influence the clinical success of the treatment [7, 10–13]. Healthcare professionals can teach, review, and correct inhaler techniques and thus improve adherence by providing support and educating their patients. However, in recent years, numerous inhalers have appeared on the market, complicating the clinician’s tasks when selecting the best inhaler for each patient [7].

The analysis of patient-reported outcomes (PROs) is being used increasingly in clinical research. The parameters consist of a patient’s evaluation of a drug based on their own perception of their disease and treatment(s). Since the device itself and its suitability for the patient are important elements to consider [14], strategies are needed to improve public health issues and outcomes in individuals with asthma and/or COPD [15]. There is currently little evidence to determine how handling errors and PROs for DPIs influence the effectiveness of these therapies. The aim of this review was to analyze the impact of PROs and handling errors in the use of Spiromax^®^, Turbuhaler^®^, and Easyhaler^®^ on asthma and COPD control, in order to guide the selection of the best DPI for each patient.

## DPIs: Spiromax^®^, Turbuhaler^®^, and Easyhaler^®^, Inhalation Features and Techniques

The dose delivered by an DPI and the particle size distribution are determined by the formulation, the inhaler, and the patient’s inhaler technique [16]. This technique depends on a wide variety of factors, such as acceleration at the beginning of inhalation (ACC), peak inspiratory flow (PIF), and total inhaled volume (IV), which are all different for each DPI [17, 18]. A growing body of evidence suggests that a correct inhaler technique is essential if treatment is to be effective. Moreover, device type and mastery of the technique play an important role in improving adherence, clinical outcomes, quality of life, and the use of health resources [13].

The ICS/LABA fixed-dose combination for the BF formulation has now been approved in Europe for 3 different DPIs: Spiromax^®^, Turbuhaler^®^, and Easyhaler^®^. A review by Haidl et al. [19] comparing inhaler techniques among the different DPIs found that Spiromax^®^, Turbuhaler^®^, and Easyhaler^®^ required a minimum inspiratory flow rate of 30 L/min for an acceptable inhalation. Flow resistance is medium-to-high for Spiromax^®^ and Turbuhaler^®^ and high for Easyhaler^®^. For all 3 devices, dose delivery is flow-dependent and guidelines recommend exhalation into the room (not into the device) and forced inhalation through the mouth from the very beginning of the maneuver [19]. In an in vitro study, Spiromax^®^ was more consistent than Turbuhaler^®^ in a range of inspiratory flow profiles, and the delivery of fine-particle doses in weak inhalation profiles tended to be higher [16].

## Inadequate Disease Control: Errors in Inhaler Technique, Lack of Adherence, and other Limitations in the Use of DPIs

Although DPIs are appropriate for the management of asthma and COPD [7], patients often present persistent symptoms, a loss of disease control, and a diminished quality of life [20]. The design of the inhaler itself can contribute to patient errors in the maneuver [21–23]. Published data also suggest that a large proportion of individuals using DPIs develop a suboptimal inhaler technique [24–26].

Evidence gathered from several studies in routine clinical practice in patients with asthma and COPD has shown that incorrect inhaler use is associated with poor symptom control and worse outcomes [8, 24, 27]. An inadequate inhaler technique in asthmatic patients, in particular, results in poor disease control [8, 13, 24], and these patients often use their DPI incorrectly [27–29]. The risk of death due to asthma or COPD increases significantly when treatment is not maintained over time [30].

Lack of therapeutic adherence ranges between 40 and 78% in asthma and 40% and 60% in COPD [31, 32]. This variability depends, to a large extent, on our definition of adherence and how it is measured. Frequency, complexity, and duration of treatment are some of the factors that can affect adherence [33]. Other factors that can limit the effectiveness of inhaled therapy include less time dedicated to training, and patient characteristics such as level of training, age, and level of education [34].

## PROs in Inhaled Therapy for Chronic Respiratory Diseases

PROs provide unique information from the patient’s perspective on how a treatment influences their attitude to their disease [35]. Furthermore, there are certain data that only PROs can provide [36]. These outcomes are becoming increasingly important in clinical research [36] to the extent that nowadays many studies include validated PRO questionnaires among their study variables [35]. In particular, current recommendations on asthma and COPD management encourage active patient participation in the selection of an inhaler device [7, 15].

## Clinical Development with Spiromax^®^, Turbuhaler^®^, and Easyhaler^®^

Although the clinical importance of the BF combination in inhaled treatment of asthma and COPD is well established, its effectiveness depends on patients using their inhaler properly and in a sustained manner over time. However, limited information is available on the differences between existing inhalers in terms of ease of use, intuitive use, and the steps required to master the technique (absence of inhaler errors) and patients’ preference for the type of inhaler prescribed to them. Studies that identify the advantages and disadvantages of each DPI in terms of inhaler technique or handling errors, patient adherence, and preferences, are therefore of the utmost importance in implementing appropriate strategies and improving inhaled therapy in patients with asthma and COPD.

## Clinical Trials with Spiromax^®^, Turbuhaler^®^, and Easyhaler^®^

A single-site, single-visit, randomized, crossover trial of 120 healthy volunteers conducted in Finland (the FINHALER study [37]) assessed device mastery, handling errors, and preferences among Spiromax^®^, Turbuhaler^®^, and Easyhaler^®^ devices. Each device was tested in 3 steps, as follows: step 1 – intuitive use; step 2 – after reading the patient information leaflet; and step 3 – after receiving instructions from their healthcare provider. Spiromax^®^ was used correctly by 37.5% and 93.3% participants in steps 1 and 2, respectively, compared with 0% and 58.3% for Easyhaler^®^, and 9.2% and 76.7% for Turbuhaler^®^. All 3 devices showed high mastery (> 95%) in step 3. The most common errors reported with Spiromax^®^ were orientation of the device, not shaking the device in the case of Easyhaler^®^, and not priming the device in the case of Turbuhaler^®^. Spiromax^®^, Easyhaler^®^, and Turbuhaler^®^ were rated as the “easiest device to use” by 73.1%, 12.6%, and 14.3% of individuals, respectively. The use of all devices improved after patients received instructions from the healthcare provider. The authors concluded that mastery of Spiromax^®^, including ease of use and intuitive use, was superior to that demonstrated for its comparators in healthy volunteers [37].

Another single-site, single-visit, randomized, crossover trial conducted in Sweden in 117 healthy adult volunteers evaluated the appropriate use of and preferences for Spiromax^®^, Turbuhaler^®^, and Easyhaler^®^. More participants mastered device handling with Spiromax^®^ than with Turbuhaler^®^ or Easyhaler^®^, both intuitively (44%, 0%, and 10%, respectively) and after reading the instructions (99%, 56%, and 81%, respectively). Fewer participants had ≥ 1 handling error with Spiromax^®^ than with the other DPIs. The percentage of volunteers still making inhaler errors after reading the instructions was 21% for Spiromax^®^ and 40% for Easyhaler^®^. Mastery of handling and inhaler techniques was lower among participants older than 60 years of age across all devices. Most individuals preferred Spiromax^®^ over Turbuhaler^®^ or Easyhaler^®^ for device handling (59%) and intuitiveness/ease of use (61%). These results highlight important differences among DPIs, which could have implications for disease control when selecting a device for a patient [38].

The ELIOT study was a 12-week, multicenter, randomized, open-label, crossover trial in 2 parallel groups conducted in the UK. The objective was to determine mastery of the inhaler technique with BF Spiromax^®^ versus BF Turbuhaler^®^ and its maintenance over time in adults with moderate–severe asthma. A total of 493 patients participated in the crossover phase (switching phase where each study group changed to the other inhaler after the initial phase), and 395 patients participated in the longitudinal phase. In the crossover phase, more patients achieved device mastery after 3 training steps with BF Spiromax^®^ (94%) versus BF Turbuhaler^®^ (87%) (odds ratio [OR] 3.77 [95% CI 2.05–6.95]; *p* < 0.001). Asthma control improved in both groups according to the Asthma Control Questionnaire (ACQ) (OR 0.11 [95% CI − 0.09 to 0.30]), but differences were not significant. An exploratory analysis by independent experts indicated that the probability of maintaining device mastery after 12 weeks was significantly higher for BF Spiromax^®^ versus BF Turbuhaler^®^ (OR 2.11 [95% CI 1.25–3.54]). Median Patient Satisfaction and Preference Questionnaire (PSAPQ) scores were higher for BF Spiromax^®^ versus BF Turbuhaler^®^ (89.8 vs. 85.7; *p* < 0.001). According to the ACQ, disease control improved with both DPIs, although changes were non-significant (OR 0.11 [95% CI − 0.09 to 0.30]) [39].

In another phase 3b, 12-week, multicenter, randomized, double-blind, double-dummy trial, the efficacy and safety of BF Spiromax^®^ versus BF Turbuhaler^®^ was assessed in 574 patients older than 12 years of age with persistent asthma. Morning peak expiratory flow (PEF), patient satisfaction, and preference were analyzed using validated questionnaires, along with other variables, including safety. Based on PEF figures, the non-inferiority of BF Spiromax^®^ versus BF Turbuhaler^®^ was demonstrated in asthmatic patients over 12 years of age. More individuals preferred BF Spiromax^®^ over BF Turbuhaler^®^ for its performance, and were willing to continue with BF Spiromax^®^ beyond the study period [40].

Finally, an open-label, cross-sectional trial in 61 patients with asthma and 44 with COPD conducted in Germany evaluated 10 different types of placebo inhalers. These included Spiromax^®^, Turbuhaler^®^, and Nexthaler^®^, which were used randomly. Spiromax^®^ required fewer attempts to ensure correct use compared with the average observed for all 10 devices (1.22). The device with the lowest mean number of attempts to error-free use was the Turbuhaler^®^ (1.02), followed by the Nexthaler^®^ (1.04), Diskus^®^ (1.07), and the Spiromax^®^ (1.10). Overall, 41% of subjects chose one of the devices they already used as their preferred inhaler. In total, 20% opted for the Spiromax^®^, 15% for the Nexthaler^®^, and 14% for the Turbuhaler^®^ or a pressurized metered dose inhaler (pMDI). Patients stated that the most important feature of an inhaler is easy handling, followed by a short inhalation time and low inhalation resistance. The authors concluded that patient preferences may vary among inhalers. The lowest number of attempts to achieve error-free use was reported for the Turbuhaler^®^ and Nexthaler^®^ devices. Overall, Spiromax^®^ and Nexthaler^®^ achieved the best overall ratings and where the devices most preferred by patients [41], 1.02 should be replaced by 1.10. Table 1 lists clinical trials conducted with the Spiromax^®^, Turbuhaler^®^, and Easyhaler^®^ devices.

**Table 1:** Clinical trials reporting PROs, handling errors, and disease control with Spiromax^®^

**Study**	**Design**	**Population**	**Duration**	**Objective**	**No. patients**	**Main findings**
Sandler et al. [37]	Single-center, crossover	Healthy volunteers	Single visit	To assess mastery, handling errors and preferences among Spiromax^®^, Turbuhaler^®^ and Easyhaler^®^	*N* = 120 (20 in each of the 6 groups)	Spiromax^®^ was the easiest DPI to use (73.1%), followed by Turbuhaler^®^ (12.6%) and Easyhaler^®^ (14.3%).
Rönmark et al. [38]	Single-center, randomized, crossover	Healthy volunteers	Single visit	To assess proper use and preferences of Spiromax^®^, Turbuhaler^®^ and Easyhaler^®^	*N* = 117	Spiromax^®^ was preferred over Turbuhaler^®^ and Easyhaler^®^ by most patients in terms of device handling (59%) and intuitive use/ease of use (61%).
Price et al. [39]	Multicenter, randomized, open-label, parallel-group, crossover	Adults with moderate-to-severe asthma	12 weeks	To determine inhaler technique and achievement and maintenance of mastery BF Spiromax^®^ and BF Turbuhaler^®^	Crossover *N* = 493 Longitudinal *N* = 395	BF Spiromax^®^ showed higher levels of DPI mastery than BF Turbuhaler^®^ (OR 2.11 [95% CI 1.25–3.54], *p* = 0.005).
						BF Spiromax^®^ was preferred over BF Turbuhaler^®^ according to PASAPQ.
						Asthma control improved with both groups according to the ACQ, (OR 0.11 [95% CI − 0.09 to 0.30], *p* = 0.278).
Virchow et al. [40]	Multicenter, phase 3b, randomized, double-blind, double-dummy	Patients ≥ 12 years with persistent asthma	12 weeks	To evaluate the efficacy and safety of BF Spiromax^®^ vs. BF Turbuhaler^®^	*N* = 574	BF Spiromax^®^ demonstrated non-inferiority vs. BF Turbuhaler^®^ according to PEF.
						BF Spiromax^®^ was preferred by a greater number of patients.
						More patients wanted to continue with BF Spiromax^®^ after the study.
Schreiber et al. [41]	Single-center, open-label, cross-sectional	Adults with asthma or COPD	Single visit	To evaluate the correct use and preference of 10 inhalers	Asthma *N* = 61 COPD *N* = 44	Spiromax^®^ was preferred by 20% of patients, followed by Nexthaler^®^ (15%) and Turbuhaler^®^ (14%) or a pMDI, according to PASAPQ and FSI-10.

*DPI* dry powder inhaler, *BF* budesonide and formoterol, *OR* odds ratio, *CI* confidence interval, *PASAPQ* patient satisfaction and preference questionnaire, *ACQ* asthma control questionnaire, *PEF* peak expiratory flow, *COPD* chronic obstructive pulmonary disease, *pMDI* pressurized metered dose inhaler, *FSI-10* Feeling of Satisfaction with inhaler questionnaire

## Real-Life Experience: Observational Studies with Spiromax^®^, Turbuhaler^®^, and Easyhaler^®^

In addition to the findings of clinical trials, the outcomes of therapies in routine clinical practice need to be analyzed and understood, as these parameters contribute to clinical decision-making.

Thus, in a prospective, observational, multicenter study conducted in Spain and Portugal as part of an international program, handling and inhaler errors with BF Spiromax^®^ and BF Turbuhaler^®^ were evaluated in 175 patients with asthma and COPD. The authors concluded that the total number of errors (1.4 vs. 1.9; *p* < 0.001) and handling errors (0.5 vs. 0.8; *p* < 0.001) per patient was significantly lower with BF Spiromax^®^ than with BF Turbuhaler^®^. BF Spiromax^®^ was easier to learn to use (*p* < 0.001), easier to prepare (*p* < 0.001), and more comfortable in terms of weight and size (*p* < 0.001). Furthermore, patients who used BF Spiromax^®^ felt that they were using the inhaler correctly (*p* < 0.001). In addition, 79.5% of the subjects preferred BF Spiromax^®^ over BF Turbuhaler^®^ [15].

The SPRINT study is a phase 4, multinational, prospective, observational study conducted in 10 European countries that evaluated the effect of a fixed-dose combination of ICS/LABA in patients with asthma and COPD. As part of the secondary objectives, an exploratory analysis was designed to assess the adherence, satisfaction, and ease of use of BF Spiromax^®^ in routine clinical practice during a visit in a cross-sectional study. Of the 1101 patients included, 342 were receiving treatment with BF Spiromax^®^. Of these, 235 had asthma and 107 had COPD. Overall, 72.5% of BF Spiromax^®^ users showed medium-to-high adherence on the Morisky Medication Adherence Scale (MMAS-8 score > 6). The mean satisfaction score for BF Spiromax^®^ on a scale of 1 to 10 was 8.9 (standard deviation [SD] 1.6). Nearly all (98.8%) of the BF Spiromax^®^ users were at least satisfied with their inhaler, while 85.4% were very satisfied. Mean ease-of-use score for BF Spiromax^®^ was 9.1 (SD 1.3). In conclusion, asthma and COPD patients using BF Spiromax^®^ showed moderate-to-high therapeutic adherence, were very satisfied with their inhaler, and found it easy to use [42].

A national multicenter observational study (the INHALA ZS study) that evaluated clinical efficacy, disease control, and satisfaction with BF Spiromax^®^ versus BF Turbuhaler^®^ in asthma and COPD patients was recently conducted in Spain. A total of 91 adults (66 with asthma and 25 with COPD) were selected from 2 primary care centers. All patients had been receiving treatment at baseline with BF Turbuhaler^®^ continuously for ≥ 3 months. Of these 91 subjects, 53 who showed therapeutic failure, lack of adherence, or non-compliance began treatment with BF Spiromax^®^, while 33 continued treatment with BF Turbuhaler^®^. The authors concluded that BF Spiromax^®^ was superior to BF Turbuhaler^®^ in terms of disease control in asthma patients measured with the Asthma Control Test (ACT), with an absolute mean effect at 3 months of 3.3 [95% CI − 0.4 to 2.8], *p* < 0.001. In the COPD cohort, disease control was measured with the COPD Assessment Test (CAT), which showed an absolute mean effect at 3 months of 4.34 [95% CI − 0.4 to 2.8], *p* < 0.001. Similarly, BF Spiromax^®^ was superior in terms of satisfaction (Feeling of Satisfaction with Inhaler-FSI-10 questionnaire) (mean absolute effect in asthma 9.5 [95% CI 6.4–12.6], *p* < 0.001, and mean absolute effect in COPD of 10.4 [95% CI 6.87–14.01], *p* < 0.001). BF Spiromax^®^ also showed non-inferiority to BF Turbuhaler^®^ in clinical efficacy according to forced expiratory volume in 1 s (FEV_1_) [43, 44].

Another retrospective cohort study in routine clinical practice conducted in the UK collected data from adults with asthma and COPD treated with inhaled ICS/LABA. The researchers assessed the non-inferiority of BF Spiromax^®^ after switching from another inhaler compared with continuing the original inhaler, according to a risk management algorithm. In the 385 subjects who switched to BF Spiromax^®^ (253 with asthma and 132 with COPD), non-inferiority was observed compared to the 1091 subjects who did not switch (743 with asthma and 348 with COPD) (non-significant difference + 6.6%; [95% CI − 0.3 to 13.5]). Asthma patients who switched to BF Spiromax^®^ compared with those who continued BF Turbuhaler^®^ reported fewer exacerbations (risk ratio [RR] 0.76; [95% CI 0.60–0.99] *p* = 0.044), were less likely to use high daily doses of short-acting β2 agonists (SABA) (odds ratio [OR] 0.71; [95% CI 0.52–0.98; *p* = 0.034]), used fewer SABA inhalers (RR 0.92; [95% CI 0.86–0.99]; *p* = 0.019), and were more likely to achieve treatment stability (OR 1.44; 95% CI 1.02–2.04; *p* = 0.037). There were no significant differences in these variables in COPD patients. The authors concluded that the use of BF Spiromax^®^ in routine clinical practice by patients with asthma and COPD was not inferior to BF Turbuhaler^®^ in terms of disease control. Asthma patients who switched to BF Spiromax^®^ showed reduced exacerbations, lower SABA use, and greater treatment stability compared with those who continued BF Turbuhaler^®^ [45, 46].

A 12-week prospective observational study conducted in Germany also assessed satisfaction, handling errors, disease control, and safety in adults with asthma and COPD treated with BF Spiromax^®^. Overall, 3943 patients were included, 2707 (68.7%) of which had asthma and 1236 (31.3%) COPD. At the start of the study, according to the Satisfaction with Inhalers and Preference questionnaire, 60.1% of the patients were satisfied-to-very satisfied with their previous inhaler, and this increased to 88.8% after using BF Spiromax^®^. Overall, 62.1% of pre-treated patients preferred BF Spiromax^®^ over their old inhaler. According to the modified Easy Low Instruction Over Time checklist, the rate of handling errors observed with BF Spiromax^®^ at week 12 was lower than at baseline (11.9% vs. 25.5% of the patients, respectively). After 12 weeks, 77.4% showed improved (minimally, much, or very much) overall health status compared to baseline. The severity of the disease assessed by both clinicians and patients improved during the study in both patients with asthma and COPD. BF Spiromax^®^ was well tolerated. In conclusion, BF Spiromax^®^ was associated with high satisfaction, a low handling error rate, and an improvement in clinical outcomes in patients in routine clinical practice [47].

Finally, a prospective, multicenter, observational study was conducted in France in 1435 adults with asthma who switched from their previous inhaler, BF Turbuhaler^®^ or Seretide^®^ Diskus^®^ (fluticasone/salmeterol propionate), to BF Spiromax^®^. After 12 weeks of use, inhaler techniques and the relationship between critical errors and disease control were assessed, and factors related to handling errors were identified. At the end of the study, 67% of patients were using BF Spiromax^®^ without handling errors and 88% without critical handling errors. In general, the presence of comorbidities was associated with handling errors, while concurrent illness that could affect device management and prior training was associated with critical handling errors. Most patients (85.4%) preferred BF Spiromax^®^ over their previous device. Inadequately controlled or uncontrolled asthma levels were reduced from baseline in patients using BF Spiromax^®^ (8.6% vs. 64.6%), and were higher in individuals with critical handling errors. In conclusion, effective patient training, correct inhaler technique, adherence, and devices associated with high patient satisfaction are interrelated factors key to the successful delivery of inhaled therapy in this disease. In addition, inhaler technique and patient satisfaction with the device should be routinely assessed in patients treated with uncontrolled asthma [48]. Table 2 lists observational studies in routine clinical practice conducted with the Spiromax^®^, Turbuhaler^®^, and Easyhaler^®^ devices.

**Table 2:** Observational studies reporting PROs and handling errors with Spiromax^®^, Turbuhaler^®^, and Easyhaler^®^

**Study**	**Design**	**Population**	**Duration**	**Objective**	**No. patients**	**Main findings**
Giner et al. [15]	Observational, multicenter, international study	Adults with asthma or COPD	Single visit	To evaluate the appropriate use and PROs of BF Spiromax® and BF Turbuhaler^®^	*N* = 175	• BF Spiromax^®^ showed a lower total number of errors vs. BF Turbuhaler^®^ (1.4 vs. 1.9; p < 0.001) and fewer handling errors (0.5 vs. 0.8; *p* < 0.001), respectively.
						• BF Spiromax^®^ is easier to learn how to use (*p* < 0,001), easier to prepare (*p* < 0,001), and comfortable in terms of weight and size (p < 0,001) and more patients felt that they were using the inhaler correctly (*p* < 0.001).
						• BF Spiromax^®^ was preferred by a greater number of patients (79.5%) over BF Turbuhaler^®^.
van der Palen et al. [42]	Observational, multicenter, international, cross-sectional study	Adults with asthma or COPD	Single visit	To evaluate adherence, satisfaction, and ease of use of BF Spiromax^®^	*N* = 1101	• Overall, 72.5% showed medium-to-high adherence (MMAS-8 ≥ 6).
						• Satisfaction was 8.9 (± 1.6) (scale from 1 to 10), with 98.8% being satisfied and 85.4% very satisfied.
						• Ease of use was 9.1 (± 1.3) (scale 1 to 10).
Piedra Castro et al. [43], De Simón Gutiérrez et al. [44]	Observational, multicenter, national study	Patients with asthma and COPD	3 months	To evaluate the efficacy, disease control, and satisfaction of BF Spiromax^®^ vs. BF Turbuhaler^®^	*N* = 91	• BF Spiromax^®^ was superior in terms of disease control (ACT absolute mean effect 3.3 [95% CI − 0.4 to 2.8] *p* < 0.001 and CAT absolute mean effect − 4.34 [95% CI − 0.4 to 2.8], *p* < 0.001).
						• BF Spiromax^®^ was superior in terms of satisfaction: FSI-10 (in asthma, mean absolute effect 9.5 [95% CI 6.4–12.6], *p* < 0.001, and in COPD mean absolute effect of 10.4 [95% CI 6.87–14.01], *p* < 0.001).
						• BF Spiromax^®^ showed non-inferiority vs. BF Turbuhaler^®^ in clinical efficacy according to FEV_1_.
Voorham et al. [45]	Observational, cohort, retrospective, case-matched study	Adults with asthma and COPD	1 year	To evaluate non-inferiority in disease control after switching to BF Spiromax^®^ vs. continuing with BF Turbuhaler^®^	*N* = 385 Asthma *N* = 253 COPD *N* = 132	• BF Spiromax^®^ was not inferior to BF Turbuhaler^®^ in disease control (difference + 6.6%; [95% CI − 0.3 to 13.5]).
						• Asthma patients using BF Spiromax^®^ reported fewer exacerbations (RR 0.76; [95% CI 0.60–0.99] *p* = 0.044); were less likely to use high daily doses of SABA (OR 0.71; [95% CI 0.52–0.98; *p* = 0.034]); used fewer SABA inhalers (RR 0.92; [95% CI 0.86–0.99]; *p* = 0.019); and were more likely to achieve treatment stability (OR 1.44; 95% CI 1.02–2.04; *p* = 0.037).
						• No significant differences were observed in COPD.
Gillissen et al. [47]	Prospective observational study	Adults with asthma and COPD	12 weeks	To evaluate satisfaction, errors, disease control, and safety in patients who switched to BF Spiromax^®^	*N* = 3943 Asthma *N* = 2707 COPD *N* = 1236	• The proportion of patients who were satisfied or very satisfied increased from 60.1 to 88.8%.
						• A total of 62.1% of patients preferred BF Spiromax^®^ over their old inhaler.
						• Fewer handling errors were observed with BF Spiromax^®^ (11.9% vs. 25.5%) vs. their old inhaler.
						• The health status of 77.4% of patients improved.
						• Disease severity improved according to clinicians and patients.
Roche et al. [48]	Prospective multicenter observational study	Adults with asthma	12 weeks	To evaluate inhaler techniques, the relationship between critical errors and disease control, and factors related to device handling errors after switching to BF Spiromax^®^	*N* = 1435	• Overall, 67% of patients used BF Spiromax^®^ without handling errors and 88% without critical handling errors.
						• Comorbidities were associated with handling errors, while concurrent illness that could affect device handling and prior training were associated with critical handling errors.
						• Most patients (85.4%) preferred BF Spiromax^®^ over their previous device (BF Turbuhaler^®^ or Seretide^®^ Diskus^®^).
						• Levels of inadequately controlled-uncontrolled asthma were lower in patients using BF Spiromax^®^ (8.6% vs. 64.6%), but higher in the presence of critical handling errors.

*COPD* chronic obstructive pulmonary disease, *PRO* patient-reported outcome, *BF* budesonide and formoterol, *MMAS-8* 8-item Morisky medication adherence scale, *ACT* asthma control test, *CI* confidence interval, *CAT* COPD assessment test, *FSI-10* feeling of satisfaction with inhaler questionnaire, *FEV*_*1*_ forced expiratory volume in 1 s, *RR* risk ratio, *SABA* short-acting beta-agonist, *OR* odds ratio

## Conclusions: The Search for an Ideal DPI for the Management of Asthma and COPD

In the clinical trials under review, most individuals preferred Spiromax^®^ and found it easier to use than its competitors. In asthma patients, favorable PROs were accompanied by increased disease control associated with the use of BF Spiromax^®^.

According to the observational studies analyzed, Spiromax^®^/BF Spiromax^®^ demonstrated lower handling error rates, greater preference for use, and greater adherence compared to other inhalers in patients with asthma and COPD. In terms of disease control, BF Spiromax^®^ proved to be superior or non-inferior to its competitors.

Since patient preferences may vary among DPIs, patient choices should be considered when selecting a device, in order to optimize adherence and control of asthma and COPD. Only a good patient-inhaler combination will improve the effectiveness of treatments and medical care in this population.
